# Health Related Quality of Life, Depression, Anxiety and Stress in Patients with Beta-Thalassemia Major

**Published:** 2015-12-10

**Authors:** M Adib-Hajbaghery, M Ahmadi, Poormansouri S

**Affiliations:** 1 Professor, Faculty of Nursing and Midwifery, Kashan University of Medical Sciences, Kashan, Iran.; 2 Lecturer in Nursing and Midwifery, Department of Nursing, Nursing and Midwifery School, Ahvaz Jundishapur University of Medical Sciences, Ahvaz, Iran.; 3 MS.c student in Nursing, Department of Nursing, Nursing and Midwifery School, Kashan University of Medical Sciences, Kashan, Iran.

**Keywords:** Anxiety, Beta-Thalassemia Major, Depression, Stress, Quality of life

## Abstract

**Background:**

Awareness of factors associated with quality of life (QOL) in patients with beta-Thalassemia major (β-TM) is necessary to develop clinical programs in order to improve social support and QOL in β-TM patients. This study aimed to examine QoL, depression, anxiety, and stress in β-TM patients in Ahvaz, Iran.

**Materials and Methods:**

A cross-sectional study was conducted on173 β-TM patients aged ≥12 years (12-18=55, ≥19=118). Subjects were selected using a census method. Data collection instrument consisted of three parts including: demographic questions, SF-36 questionnaire and depression, anxiety, and stress scale (DAS-21).

**Results:**

The participants obtained a mean score of 64.38±18.20 for QOL, 6.4±5.1 for depression, 4.8±3.9 for anxiety, and 7.3±4.9 for stress. Significant relationship was found between QOL and employment (P=0.02) and education level (P<0.001). Patients in the age group of 12-18 years old had higher mean scores in the majority of QoL dimensions than those aged ≤19. The mean scores of depression, anxiety, and stress were higher in patients aged ≤19. No significant correlation was observed between QOL and depression, anxiety, stress scores, and other demographic variables. Moreover, a significant inverse correlation was found between QOL and depression (P<0.001,r= -0.62), anxiety (P<0.001,r= -0.55), and stress scores (P<0.001, r= -0.5) .

**Conclusion:**

This study showed that β-TM patients experienced a considerable decrease both in their overall QoL and in its dimensions. A majority of the β-TM patients were also suffered from mild to severe depression, anxiety, and stress.

## Introduction

Beta-thalassemia major (β-TM) is an autosomal recessive congenital hemoglobinopathy. It results in a severe anemia that requires regular blood transfusions and iron chelation therapy (1). Thalassemia is the most common genetic disease in the world and in Iran (2). It is a serious problem in the eastern Mediterranean region, especially Southeast Asia and the Indian subcontinent. There 

are approximately 300 million patients with thalassemia worldwide, out of which, 55 million live in Southeast Asia (3). There are about 15000 patients with β-TM in Iran, with the highest incidence in Mazandaran, Fars, and Khuzestan provinces, respectively (4). Patients with β-TM suffer from a wide range of physical and psychological problems. Growth retardation, delayed puberty, and deformities since the disease change the patients’ physical appearance and body image which consequently lead to a poor self-image and low self-steam (3). These patients also suffer from many limitations and potentially life-threatening conditions that substantially interfere with their education and social activities (1).

Due to the advancements in medications and iron chelation therapy, there has been a steady increase in the life expectancy and survival rates of patients with β-TM in recent decades (1, 5-6). However, many patients with β-TM still report decrements in their functions, performances, and quality of life (QoL) (5-6). In addition to physical problems and functional limitations, these patients suffer from problems in marriage, education, and finding an appropriate job that not only decrease their QoL and psychological wellbeing (7,8, 9), but also affect the patient’s family and the country's healthcare system. Patients with β-TM suffer from psychological problems such as anxiety, depression, hopelessness, and difficulties in social relations (2). The psychological burden of the disease, influence on many aspects of life such as education, physical activity, skills, and fertility (1).

In a study by Sachdeva et al. a majority of patients with β-TM suffered from emotional and social problems and over 90% of patients reported low QoL (10). In another study, Thavorncharoensap et al. have reported that factors such as patient’s age, age at onset of anemia, age at first transfusion, pretransfusion hemoglobin level, receiving a blood transfusion during the last three months, and disease severity could significantly affect the QoL in Thai children with β-TM (3). Mikell and Tsiantis have also compared the depressive symptoms and QoL among healthy adolescents and those with β-TM and reported that adolescents with β-TM suffer from more depression symptoms and lower levels of QoL (11). Similar findings have also reported by Khani et al. who studied the QoL of a sample of patients with beta-thalassaemia major lived in north of Iran (12). In contrast, Hadi et al. compared the QOL of healthy adolescents and those with β-TM and did not found any significant difference between two groups in terms of happiness and vitality, sadness, weakness and fatigue (7). Poor movahed et al. have also investigated the level of hopelessness in adolescents with β-TM and found no significant difference between adolescents with β-TM and healthy controls (13). 

QoL is one of the topics that attract the attention of psychologists and human science, and health researchers in the last two decades. WHO defines Quality of Life as "individual’s perception of their position in life in the context of the culture and value systems in which they live and in relation to their goals, expectations, standards and concerns". (14). It also reflects the range of subjective human needs and shows the individuals’ and groups’ understanding of their level of wellbeing (2). QoL is also a concept related to the outcome of healthcare (including nursing care). Therefore, nurses have always tried to improve their patients QoL through providing quality care, participating in nursing research and developing caring models based of continues care and follow up (15). Though several studies are available on the QoL in patients with β-TM, few studies were performed on the factors affecting the QoL in these patients. Improving the QoL of people with β-TM and formulating health and social policies in order to improve their QoL and proper planning for the prevention, diagnosis, and timely treatment of their problems require knowledge of factors affecting the QoL in these patients. Therefore, this study aimed to examine the QoL, depression, anxiety, and stress in patients with β-TM in Ahvaz, Iran.

## Materials and Methods

A cross-sectional study was performed at the β-TM Clinic of Shafa Hospital affiliated with Ahvaz Jundishapur University of Medical Sciences from 21 April to 21 June 2014. The study population consisted of all β-TM patients over the age of 12 years old (12-18 and ≥19) referred to this center. Sampling was performed using a census method. Then, all patients with β-TM who were at age 12-18 and ≥19, received blood transfusion on a monthly basis and admitted to this center during the study were recruited. In total, 225 eligible patients were admitted to the β-TM clinic during the study; however, 27 ones were excluded due to the lack of consent and 198 patients were entered the study. 

**Instruments**

A three part instrument was used in this study. The first part was consisted of 19 questions about socio-demographic characteristics (i.e. the patient’s age, gender, marital status, education level, occupation, monthly income, ethnicity, existing of comorbidities, and complications and adherence with iron-chelation therapy). The second part of the instrument was the SF-36 questionnaire and the third part was the depression, anxiety, and stress scale (DASS 21). 

The SF-36 questionnaire consists of eight subscales of physical functioning (PF), role limitations due to physical problems (RP), bodily pain (BP), general health (GH), vitality (VT), social functioning (SF), role limitations due to emotional problems (RE), and mental health (MH). For each scale, the raw scores are transformed to 0–100 scale, with higher scores indicating better QoL. The SF-36 questionnaire was previously translated into Persian language by Montazeri et al. and showed appropriate psychometric properties. They also confirmed the instrument’s content validity and internal consistency (α= 0.77-0.9) (16). This questionnaire was also validated for implementation in patients with β-TM by Jafari et al. (17). 

The DASS 21 is a 21-item scale which includes three self-report subscales to measure the negative emotional states of depression, anxiety, and stress. Each of the three subscales contains 7 items. In completing the DASS 21, the individual is required to indicate the presence of the symptoms over the previous week. Each item is scored from 0 (did not apply to me at all over the last week) to 3 (applied to me very much or most of the time over the past week). The total score obtained for each scale was summed. Defined cutoff points are as follows: for depression score 0-4 regarded as normal, 5-6 as mild, 7-10 as moderate, 11-13 as severe and higher than 14 as extremely sever. For anxiety score, 0-3 regarded as normal, 4-5 as mild, 6-7 as moderate, 8-9 as severe, and higher than 10 as extremely severe. In terms of stress score, 0-7 regarded as normal, 8-9 as mild, 10-12 as moderate, 13-16 as severe, and higher than 17 as extremely severe. The DASS 21 is a standard tool with the internal consistence of 0.81, 0.73, and 0.81 for depression, anxiety, and stress subscales; respectively, based on a Cronbach's alpha (18). The DASS-21 scale was translated into Persian by Sahebi et al. and its appropriate content validity and psychometric properties were revealed. Its internal consistency was assessed using Cronbach's alpha and was 0.7, 0.66 and 0.76 for depression, anxiety, and stress subscales respectively (19). 

**Data collection**

One of the researchers, presented in the β-TM clinic, assessed potential participants for eligibility, briefed the eligible subjects on the study purposes, and performed the data collection. 

Patients with an education level of intermediate school and over were asked to complete the questionnaire themselves in a calm and private room in the respected clinic. However, individual interviews in a private room were used to fill out the questionnaire for those who were illiterate or at the level of elementary school. In these cases, the researcher that was trained in this regard read the questions and marked their responses in the questionnaires. 

**Ethical considerations**

The Institutional Review Board and the Research Ethics Committee of Kashan University of Medical Sciences and Healthcare Services (Ethic Code: 4850), Kashan, Iran, approved the study. In addition, required permissions were received from relevant authorities in Ahvaz Jundishapur University of Medical Sciences and the β-TM clinic of Shafa Hospital. All participants in the study signed a written informed consent form and were assured of the confidentiality of their personal information and absence of any constraint to participate in the study. 

**Statistical Analysis**

Data analysis was conducted using SPSS statistical software version 13. Descriptive statistics (frequency, mean, and standard deviation) were calculated. Moreover, Kolmogorov-Smirnov was used to examine the normal distribution of variables. Independent samples t-test was used to compare the mean scores of life quality, depression, anxiety, and stress in terms of dichotomous demographics and clinical variables. Pearson and Spearman correlation coefficients were calculated to examine the relationship between quality of life with depression, anxiety, and stress.

## Results

From a total of 198 patients with β -TM who entered the study, 25 were excluded due to the incomplete questionnaires. Therefore, the analysis was performed on 173 subjects out of them, 78 ones were males (45.1%) and 95 ones were females (54.9%). The mean age of the subjects was 23.34 ± 5.9 years out of them, 55 ones were 12-18 years old and 118 ones were 19≥ years old. Table I demonstrates the patients’ demographic and clinical characteristics.

The mean overall quality of life score of the subjects was 64.38 ± 18.20 and the mean scores of the eight scales of quality of life were 70.29 ± 24.59 (PF), 54.19 ± 38.03 (RP), 60.69 ± 38.66 (RE), 60.12 ± 21.32 (VT), 63.58 ± 20.57 (MH), 73.77 ± 23.02 (SF), 73.57 ± 27.14 (BP), and 60.23 ± 22.94 (GH), respectively. Table 2 presents the overall QoL and its dimensions mean scores in terms of study variables. No significant difference was found in the overall QoL mean score of the patients with different ages, genders, marital status, and adherence with Iron chelation therapy (P > 0.05). However, the mean overall QoL score was significantly higher in employed patients (P = 0.01) and those with college education (P < 0.001) when compared with those who were un-employed or those who had a lower education level. Table II also shows that patients in the age group 12-18 years old had higher mean scores in the majority of QoL dimensions than those over the age 19. However, the differences were not significant except for the dimension of GH (P = 0.04) and Phf dimension (P = 0.02). Male patients had also higher mean scores in most of QoL dimensions than female patients. However, the differences were not statistically significant except for the GH dimension (P = 0.02). Moreover, married subjects reported better QoL than single ones in overall QoL and all dimensions except for the physical functioning. However, the differences were not statistically significant (P > 0.05). Moreover, employed patients obtained higher mean scores in all QoL dimensions and the differences were statistically significant in the four dimensions of PF (P=0.01), VT (P=0.01), SF (P=0.005), and BP (P=0.009). In addition, patients with college education obtained higher mean scores in all QoL dimensions and the differences were statistically significant in the four dimensions of PF (P=0.001), RP (P=0.006), VT (P=0.008), and GH (P=0.03). In addition, patients with a good compliance with iron chelation therapy obtained higher mean scores in all QoL dimensions but the differences were not statistically significant except for the dimension of SF (P< 0.01). 

In terms of depression, anxiety and stress in patients with β-TM, results showed that the mean of depression, anxiety, and stress scores of the participants were 6.43±5.16, 4.86±3.96 and 7.36 ±4.99, respectively. 

**Figure 1:**
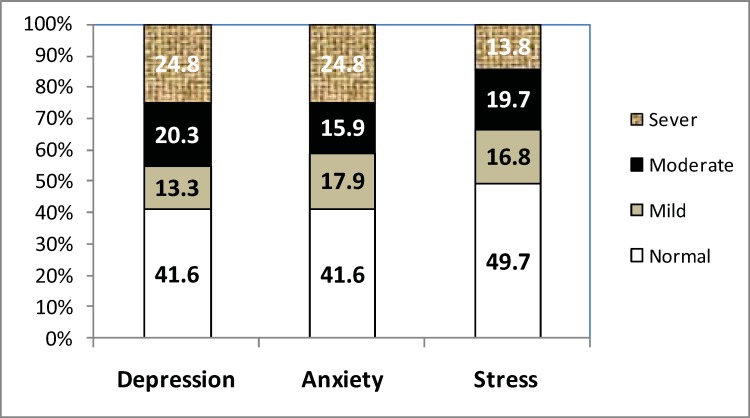
The prevalence of depression, anxiety and stress in patients with β-TM

As presented in Figure 1, 41.6% of the patients were not depressed or anxious but 58.4% suffered from mild to severe depression and anxiety. Moreover, 49.7% of the patients were in normal state of stress but 50.3% suffered from mild to severe stress.Table III shows that the mean depression score was higher in patients aged 19 years old and higher, among males, singles, and those who were unemployed, and also among patients with lower education levels and those with poor compliance with iron chelation therapy. 

However, the differences were not statistically significant. Moreover, the mean anxiety score was higher in patients aged 19 years old and higher, among singles and those who were unemployed, and also among patients with lower education levels and those with poor compliance with iron chelation therapy. However, the differences were not statistically significant except for the patients with different education levels (P < 0.04). In addition, the mean stress score was higher in patients aged 19 years old and higher, among females, singles and those who were unemployed, and also among patients with lower education levels and those with poor compliance with iron chelation therapy. However, the differences were not statistically significant except for the patients aged 19 years old and higher than those with lower ages (P = 0.008).

Headache (47.4%) and joint disorders (24.3%) were found among the most prevalent co-morbidities and complications in the study participants.

Moreover, the relationship between the presence of complications and quality of life, depression, anxiety, and stress were assessed and patients with complications obtained higher scores in, depression, anxiety, and stress while they got lower scores in QoL. In some cases the differences were significant (Table IV). 

Significant correlations were found between the patients QoL scores and their scores of depression (P<0.001,r=-0.62), anxiety (P<0.001,r=-0.55), and stress (P<0.001,r=-0.5), respectively (Table V).

**Table I:** Demographic features of participants in the study

**Demographic profile**		**frequency**	**%**
**sex**	female	95	54.9
	male	78	45.1
**Age **	12-18	55	31.8
	≤19	118	68.2
**Marital status**	Single	159	91.9
	Married	13	7.5
	Divorced	1	0.6
**Ethnicity**	Arab	77	44.5
	Fars	96	55.5
**Educational level**	Illiterate	2	1
	<High school Diploma	35	20
	High school Diploma	76	44
	College	60	35
**Employment **	Not employed	83	48
	Employed	23	13.3
	Student	67	32.4
**Income per month** **(Rial)**	5000000 >	152	87.8
	5000000-10000000	13	7.5
	10000000<	8	4.6

**Table II:** Comparison quality of life score and its dimensions in the sample Case Study are Based on demographic variables

			**Physical functioning**	**Role-physical**	**Role-emotional**	**Vitality**	**Mental health**	**Social functioning**	**Bodily pain**	**General health**	**Total SF-36**
**Variables**		N	M±SD	M±SD	M±SD	M±SD	M±SD	M±SD	M±SD	M±SD	M±SD
**Age**	12-18	55	73.14±23.45	60±36.19	69.09±35.63	61.18±21.83	64.51±18.93	78.41±23.50	76.45±26.19	65.36±21.89	65.71±18.40
	≤19	118	64.18±26.06	51.48±38.71	56.78±39.53	59.62±21.16	63.15±21.35	71.61±22.56	72.22±24.63	57.84±23.17	63.76±18.16
	P value		0.02	0.1	0.05	0.65	0.68	0.07	0.3	0.04	0.5
**Sex**	male	78	71.47±24.98	53.21±38.93	59.40±37.85	61.99±22.51	66.10±22.65	73.88±24.35	73.91±27.98	61.15±23.25	65.19±20.09
	female	95	69.32±24.36	55±37.47	61.75±39.49	58.58±20.29	61.52±18.56	73.68±21.99	73.29±22.70	59.47±22.77	63.71±16.58
	P value		0.5	0.7	0.6	0.2	0.1	0.9	0.8	0.6	0.5
**Marital status**	Single	159	70.31±24.54	53.66±38.13	59.75±38.98	59.62±21.35	62.82±20.53	73.35±23.36	73.57±24.95	59.69±22.97	63.96±18.14
	Married	13	69.62±27.11	63.46±37.66	74.36±33.75	66.54±21.54	73.54±19.83	80.77±17.39	75±28.84	68.85±21.42	70.44±19.09
	P value		0.9	0.3	0.1	0.2	0.07	0.2	0.8	0.1	0.2
**Employment**	Not employed	83	69.10±22.94	45.78±35.20	54.62±38.81	55.06±18.22	60.14±19.50	67.47±22.92	66.81±24.16	55.18±24.27	60.05±17.79
	Employed	23	80.87±19.16	70.58±38.88	59.42±37.54	65.87±20.76	68±21.06	82.61±21.23	81.74±23.12	61.74±20.20	70.25±14.80
	P value		0.01	0.1	0.5	0.01	0.09	0.005	0.009	0.2	0.01
**Educational level**	≤High school Diploma	113	65.84±24.28	48.45±38.27	59±38.32	56.99±21.10	61.73±20.03	72.01±24.79	71.73±26.32	57.48±23.35	61.15±17.57
	College	60	78.67±23.12	65±35.41	63.89±39.43	66±20.66	67.07±21.28	77.08±19	77.04±22.55	65.42±21.37	70.45±17.97
	P value		0.001	0.006	0.4	0.008	0.1	0.1	0.1	0.03	0.001
**Compliance with iron-chelating therapy**	Good	153	71.21±24.10	55.23±38.22	61.87±39.07	61.08±20.99	64.18±20.57	75.82±21.87	74.69±24.73	61.08±22.64	65.38±17.74
	Poor	17	64.71±28.42	52.94±35.22	52.94±37.37	54.41±23.97	60.94±21.42	61.76±25.56	66.03±28.91	57.06±25.25	59.19±20.85
	P value		0.3	0.8	0.3	0.2	0.5	0.01	0.1	0.4	0.3

**Table III:** Comparison depression, anxiety, stress in the sample Case Study are Based on demographic variables

			**Depression**		** Anxiety**		**Stress**	
**Variables**		N	Mean±SD	P value	Mean±SD	P value	Mean±SD	P value
**Age**	12-18	55	5.87±5.31	0.3	4.6±4.1	0.5	5.9±4.6	0.008
	≤19	118	6.7±5.09		4.9±3.8		8.05±5.01	
**Sex**	male	78	6.6±5.7	0.6	4.8±4.09	0.9	7±5.3	0.3
	female	95	6.2±4.7		4.8±3.8		7.6±4.6	
**Marital status**	Single	159	6.5±5.1	0.2	4.8±3.9	0.4	7.4±5.04	0.5
	Married	13	4.6±5.3		4.07±3.3		6.5±4.3	
**Employment**	Not employed	83	7.3±5.1	0.09	5.6±4.03	0.2	8.4±4.7	0.1
	Employed	23	5.2±4.9		4.5±3.7		6.6±5.2	
**Educational level**	≤High school Diploma	113	6.8±5.1	0.2	5.3±4.1	0.04	7.6±4.9	0.4
	College	60	5.7±5.1		4.01±3.5		6.9±5	
**Compliance with iron-chelating therapy**	Good	153	6.2±5.1	0.4	4.6±3.9	0.5	7.05±4.9	0.05
	Poor	17	7.2±5.02		5.2±3.3		9.5±5.2	

**Table IV:** relationship between disease comorbidities and quality of life, depression, anxiety,and stress

			**Total SF-36**		**Depression**		**Anxiety**		**Stress**	
**Comorbidities**		N(%)	M±SD	P	M±SD	P value	Mean±SD	P value	Mean±SD	P value
**Headache**	yes	82 (47.4)	60.75±17.54	0.01	7.1±5.1	0.08	5.3±3.8	0.1	8.2±4.6	0.02
	no	91 (52.6)	67.65±18.27		5.8±5.1		4.4±4		6.5±5.1	
**Articular Diseases**	yes	42 (24.3)	58.61±16.56	0.01	8.6±4.9	0.001	6.3±3.6	0.004	9.8±5.08	> 0.001
	no	131 (75.7)	66.23±18.38		5.7±5.03		4.3±3.9		6.5±4.7	
**Heart diseases**	yes	24 (13.9)	55.15±15.16	0.007	9.5±5.1	0.001	7.8±3.9	> 0.001	9.7±4.3	0.01
	no	149 (86.1)	65.87±18.26		5.9±5		4.3±3.7		6.9±5	
**Spleen disease**	yes	24 (13.9)	57.99±17.65	0.06	7.7±5.5	0.1	5.7±4.3	0.2	9±5	0.07
	no	149 (86.1)	65.4±18.14		6.2±5		4.7±3.9		7.1±4.9	
**Back pain**	yes	21 (12.1)	61.02±19.68	0.3	7±5.4	0.5	5.8±4.2	0.2	7.3±5	0.9
	no	152 (87.9)	64.84±18.01		6.3±5.1		4.7±3.9		7.3±4.8	
**Diabetes**	yes	16 (11)	60.50±19.58	0.01	7.2±5.6	0.001	6.4±3.8	0.06	7.9±4.8	0.5
	no	154 (89)	64.86±18.04		6.3±5.1		4.6±3.9		7.2±5.01	
**Hypothyroidism**	yes	18 (10.4)	63.38±19.92	0.8	8.1±5.1	0.1	5±3.4	0.8	8.6±4	0.1
	no	155 (89.6)	64.50±18.06		6.2±5.1		4.8±4.03		7.2±5	
**Shortness of breath**	yes	18 (10.4)	57.45±15.07	0.08	8.1±5.5	0.1	7.3±4.5	0.004	10.2±5.9	0.009
	no	155 (89.6)	65.18±18.41		6.2±5.09		4.5±3.8		7.03±4.7	
**Skin problems**	yes	17 (9.8)	53.47±19.16	0.009	10.1±5.1	0.002	7.3±3.9	0.006	9.7±4.4	0.04
	no	156 (90.2)	65.57±17.76		6.03±5.02		4.5±3.8		7.1±4.9	

**Table V:** relationship between quality of life and its dimensions with depression, anxiety, stress

	**Depression**	**Anxiety**	**Stress**
**dimensions of quality of life**	P value (r)	P value (r)	P value (r)
**physical functioning**	<0.001(-0.29)	<0.001(-0.3)	<0.001(-0.2)
**Role-physical**	<0.001(-0.4)	<0.001(-0.4)	<0.001(-0.33)
**Role-emotional**	<0.001(-0.41)	<0.001(-0.37)	<0.001(-0.39)
**Vitality**	<0.001(-0.51)	<0.001(-0.42)	<0.001(-0.53)
**Mental health**	<0.001(-0.47)	<0.001(-0.48)	<0.001(-0.6)
**Social functioning**	<0.001(-0.51)	<0.001(-0.49)	<0.001(-0.47)
**Bodily pain**	<0.001(-0.47)	<0.001(-0.42)	<0.001(-0.39)
**General health**	<0.001(-0.54)	<0.001(-0.49)	<0.001(-0.46)
**Total SF-36**	<0.001(-0.62)	<0.001(-0.55)	<0.001(-0.5)

## Discussion

This study aimed to examine the QoL, depression, anxiety, and stress in patients with β-TM. The study results showed that all aspects of the QoL were impaired in patients with β-TM especially in the dimensions of RP, RE, VT, MH, and GH. This findings revealed that patients with β-TM experienced considerable limitations in their role performances not only for their physical problems but also due to emotional problems and significant decreases in their energy level and general health. Our findings were in consistent with results of studies conducted by Haghpanah et al. in Shiraz, Safizadeh et al. in Kerman and Ansari et al. in Tehran (9,20,21). In the current study, more than half of the patients with β-TM suffered from mild to severe depression, anxiety, and stress. Moreover, a significant inverse association was found between the scores of depression, stress and anxiety and the overall QOL score. These findings were consistent with findings of Shaligram et al. who studied the relationship between psychological problems and QoL in children with thalassemia (22). Our findings were also in consistent with findings of Naderi et al. who studied mental health of patients with β-TM in south- east of Iran (23). It seems that psychological disorders are common among patients with β-TM. Previous studies have also showed that compared to the healthy people, patients with β-TM suffer from higher levels of depression and anxiety, experience a significant decrease in their psychological wellbeing and 80% of them suffer at least from one psychiatric disorder (21, 24). Moreover, the inverse correlation between the patients QoL score and their scores of depression, anxiety, and stress confirmed that psychological problems in patients with β-TM can profoundly affect their QoL. Therefore, it is needed to establish supportive systems including some psychological consultation systems at the thalassemia clinics. In the present study, patients with lower educations not only got a lower mean of QoL but also suffered from more depression, anxiety, and stress. Ansari et al. (21) and Kaheni et al. (25) have also studies the QoL of patients with β-TM and reported significant relationship between patients’ education level and their QoL. Naderi et al. have also reported a high prevalence of mood and anxiety disorders among patients with thalassemia (23). Despite advances in the process of thalassemia treatment, these patients are faced with hard life conditions, expecting death and uncertainties (23) that consequently predisposed them to depression, anxiety, and stress. It seems these psychological problems would negatively affect the patients QoL. 

In the present study, unemployed patients not only obtained a lower mean in QoL but also suffered from higher levels of depression, anxiety, and stress. Though in thalassemia, previous studies have not reported that unemployed people generally suffered from lower levels of QoL than employed ones (26). As the present study showed, patients with thalassemia suffered not only from lower levels of energy and vitality but also from much more physical pains and limitations in their physical and emotional roles that consequently interfered with their work abilities. Then, they were more appt to be evicted from their work or being unemployed. Unemployment may consequently exacerbate the patients’ depression, anxiety and stress and would consequently lead to more decrease in their QoL. 

In the present study, only 7.5% of the patients had been married. Moreover, unmarried and single patients reported worse QoL than married ones (in overall QoL and in the majority of its dimensions) though the differences were not statistically significant. Unmarried patients had also got higher (but statistically insignificant) scores in terms of depression, anxiety, and stress scale. Consistent with our findings, a previous study, showed no significant correlation between marital status and QOL (9). However, the statistically non significant results of the present study may be attributed to the small number of the married patients. Presently, the screening tests for β-TM are among the compulsive pre-marriage testes in Iran. A positive test usually stops the process of marriage. In addition, other factors such as disease complications, infertility, and dependence on blood transfusions may exacerbate the patients’ psychological symptoms and decrease their QoL. In the present study, patients in the age group over 19 years had lower mean scores not only in the overall QoL but also in majority of its dimensions (though the differences were not significant). Moreover, patients older than 19 years suffered from higher levels of depression, anxiety, and stress. In contrast, Naderi et al. found no significant relationship between age and the prevalence of psychological disorders in patients with thalassemia (23). Perhaps the reason is that in Naderi et al’s study, all patients were younger than 25 but in the present study a significant number of patients were older than 19. It seems that, problems in marriage and employment, and dealing with more responsibilities in life, led to the higher degrees of depression, anxiety and stress in these patients.

In the current study, male patients had better QoL than females, though the difference was not statistically significant. Moreover, no significant differences were observed in the mean of depression, anxiety, and stress scores in the two genders. Haghpanah et al., Kaheni et al., and Safizadeh et al. also did

not find a significant relationship between age and QOL (9,20,25). In contrast, Thavorncharoensap et al. (3) and Sobota et al. (27) reported a significant relationship between age and QoL in patients with thalassemia so that older patients had worse QoL. Naderi et al. (23) also did not find a significant relationship between gender and psychosocial wellbeing. Despite the statistically non significant results in terms of gender, the lower QoL mean scores of female patients revealed that females may be more vulnerable than males to the negative effects of the disease. 

Results of this study showed that patients with clinical complications and those with poor compliance with iron-chelation therapy had not only lower QoL mean scores but also higher scores in terms of depression, anxiety, and stress. Haghpanah et al. (9), Sobota et al. (27), and Shaligram et al. (22) have also found worse QOL in patients with β-TM who had co-morbidities and poor compliance with iron-chelation therapy (9). Yahia et al. have also studied the predictors of anxiety and depression in Egyptian thalassemic patients and found a significant relationship between the presence of complications such as heart disease, diabetes, short stature and delayed puberty with psychological problems such as depression and anxiety (24). It seems that posing co-morbidities and clinical complications may not only insert a direct effect on the QoL in patients with β-TM, but also will increase the patients’ psychological problems that consequently increase their need for additional treatments and repeated hospital admissions and eventually reduce the patients QoL. On the other hand, good adherence with iron-chelation therapy regimen may not only improve the patients’ QoL by reducing the risk and the rate of complications, but also may improve the patients’ psychological condition.

## Conclusion

This study showed that higher ages, unemployment, low education level, being single, the presence of co-morbidities and complications, poor compliance with iron chelation therapy and existing of psychological problems can negatively affect the QoL in patients with β-TM. While variables such as the patients’ age and gender cannot be modified and the patients’ education level and marital status are not easily and quickly modifiable, however, other variable may be modifiable in a multidisciplinary approach. Nurses should focus on disease specific educational programs for patients with β-TM about the entity and the process of the disease, its managements, self-care needs, importance of regular iron chelation therapy and its role in preventing or delaying complications. Moreover, nurses should collaborate with psychologist in providing counseling programs and specific anxiety and stress management programs to help patients for better adaptation with the disease specific and the daily stressors. Moreover, nurses may collaborate with some professional and social supportive and counseling agencies to help patients find appropriate jobs and alleviate some of their financial requirements. Finally, nurses may establish regular health assessment and physical examinations for patients to be done in the β-TM clinics. This may help early diagnosis of complications. Then appropriate treatment programs may be established. A combination of the aforementioned programs, if established, may help people in coping with this chronic disease and improvment of. Moreover, the effects of such programs on the patients QoL can be assessed. The current study was limited due to lack of a control group, and therefore the comparison of QoL scores of patients with β-TM with normal population was not possible. Therefore, a case- control study to compare the quality of life and psychological disorders between healthy populations and patients with β-TM is suggested. 
